# The associations between injury mechanism and extended hospital stay among pediatric patients: findings from a trauma Center in Saudi Arabia

**DOI:** 10.1186/s12887-019-1559-7

**Published:** 2019-06-03

**Authors:** Suliman Alghnam, Jawaher Ali Towhari, Ibrahim Al Babtain, Muhannad Al Nahdi, Mohammed Hamad Aldebasi, Mahna Alyami, Hamad Alkhalaf

**Affiliations:** 1 Population Health Section-King Abdullah International Medical Research Center (KAIMRC), King Saud bin Abdulaziz University for Health Sciences (KSAU-HS), Riyadh, Saudi Arabia; 2 College of Medicine, King Saud bin Abdulaziz University for Health Sciences, Riyadh, Saudi Arabia; 3 General Surgery Trauma and Acute Care Surgery, King Abdulaziz Medical City, Riyadh, Saudi Arabia; 4 College of Medicine, Imam Mohammad Ibn Saud Islamic University (IMSIU), Riyadh, Saudi Arabia; 5 Health Education Department, Saudi German Hospital, Riyadh, Saudi Arabia; 6 General Pediatrics and Complex Care, King Abdullah Specialized Children’s Hospital, King Saud bin Abdulaziz University for Health Sciences, Riyadh, Saudi Arabia

**Keywords:** Injury mechanism, Length of stay, Pediatric trauma, Saudi Arabia

## Abstract

**Background:**

A hospitalized patient’s length of stay (LOS) can have a significant impact on the performance and operating costs of a healthcare facility. Among pediatric patients, traumatic injuries are common causes of emergency room visits and hospitalizations. In Saudi Arabia, little is known about the burden of pediatric traumas on population health and the healthcare facilities. Therefore, the aim of this study was to investigate the associations between traumatic pediatric injury mechanisms and extended LOS in a trauma center.

**Methods:**

Data was obtained from the trauma registry. From 2001 to 2018, trauma patients between the ages of 0 and 18 years old with LOSs of > 0 days were analyzed. The independent variable was the injury mechanism, which was classified as follows: falls, burns, drowning, motor vehicle collisions, motorcycle collisions, pedestrian, and intentional injuries. The dependent variable was an extended LOS defined as ≥21 days. A multivariate logistic regression analysis was used to evaluate the associations between the injury mechanisms and an extended LOS.

**Results:**

A total of 5563 pediatric patients were included in this study. Of those, 774 (14%) had extended LOSs. Those patients with extended LOSs suffered more severe injuries than those with short hospital stays as measured by the Injury Severity Score (mean scores: 15.4 vs. 6.8, *p* < 0.01), the Glasgow Coma Scale score (mean scores: 10.4 vs, 14.0, *p* < 0.01), and the Revised Trauma Score (mean scores: 9.9 vs. 11.0, *p* < 0.01). Approximately one half of the patients with extended LOSs were admitted due to motor vehicle injuries. In addition, those patients were almost five times more likely to have extended LOSs than the patients who suffered fall injuries (odds ratio: 4.8, 95% confidence interval: 3.2–7.1).

**Conclusions:**

Based on the study results, motor vehicle injuries were significantly associated with extended hospitalizations. Prevention is instrumental for reducing healthcare utilization; therefore, these findings call for public health professionals and policymakers to plan, design, and implement preventive measures to reduce the traffic injury burden. In addition, increased traffic law enforcement, such as the use of car restraints, is warranted to reduce the preventable injuries and improve the overall population health.

## Introduction

Hospital quality improvement and cost reduction continue to be some of the most critical issues for healthcare payers, providers, and policymakers worldwide. A patient’s hospital length of stay (LOS) is one of many criteria that are widely used to evaluate a hospital’s performance and operating costs [1]. Inevitably, extended hospitalizations are associated with increased costs, which place a greater burden on the healthcare system [2]. Each extended hospitalization day for a single patient can cost as much as $836 US dollars, according to a study of community-acquired pneumonia patients [3]. Naturally, reducing hospital LOSs is instrumental for achieving service efficiency and minimizing the healthcare-associated infection risk.

Certain injuries are associated with the hospital LOS. Globally, previous literature has investigated the causes of extended LOSs; for example, one study in Iran found that age, gender, and injury characteristics were associated with the hospital LOS [4]. Another study compared the hospital LOSs of teaching and nonteaching hospitals, and the results showed no differences among the pediatric trauma cases [5]. Other studies have examined the factors associated with an extended LOS among pediatric patients admitted to the emergency department (ED) [6]. In one study conducted in China, several factors were associated with hospital LOSs > 24 h in pediatric emergency units, including the age, the need for emergency transfusions, and the sociodemographic conditions [7].

In Saudi Arabia (SA), injuries are a significant threat to the overall population health, especially among children. Despite this, there is limited literature in the field of pediatric hospital LOSs. Ministry of Health data has indicated that the average LOS for all conditions decreased from 4.8 days in 2012 to 4.4 days in 2016 [8]. Obtaining more information about hospital LOSs among pediatric trauma patients is instrumental to understanding the healthcare burden, resource allocation, and prevention plans.

An extended pediatric hospitalization is a traumatic and stressful event for the child and his or her family, in addition to its negative impact on healthcare utilization and costs [9]. Even though the hospital LOSs among children have decreased over the years, more children are being hospitalized for conditions that are usually associated with adults (i.e., chronic conditions) [10]. For acute conditions, such as injuries, the factors that play roles not only in admission but also in extended LOSs are less clear.

Examining the associations between the injury mechanisms and extended LOSs may help identify the most devastating injury mechanisms, and therefore, provide information for preventative efforts. In addition, such an investigation may also help improve the quality and safety of healthcare systems by determining the current and future burdens of extended hospital LOSs in order to allocate resources efficiently. Therefore, the purpose of this study was to determine the associations between the injury mechanisms and extended LOSs among pediatric patients admitted to a trauma center in SA.

## Methods

This retrospective study was conducted at the King Abdulaziz Medical City (KAMC). The only patients eligible for treatment at this facility are those working at the Ministry of National Guards and their parents, spouses, and children. However, in an emergency, any patient can be admitted and receive medical care if needed. The KAMC is considered to be an advanced trauma center because it offers trauma care 24 h a day, including immediate access to general, vascular, and orthopedic surgeons. Additionally, the KAMC is among the few hospitals in SA that are accredited by the American College of Surgeons to provide training in advanced trauma life support [11]. The KAMC has approximately 200 beds for pediatric patients, of which 60 are in the ED.

### Study population

The dataset used in this study was obtained from the KAMC trauma registry. Since its initiation in 2001, the registry has collected detailed information on all of the hospital admissions following an acute injury. The registry also captures the deaths announced at any point (including deaths upon arrival). However, those patients treated in the ED who are then discharged are not included. In the ED, the patients are triaged according to the injury severity, the medical care is provided by the relevant specialty (i.e., surgery), and the patient is admitted if necessary. An assigned coordinator inspects all of the daily admissions in order to identify trauma-related admissions. Once these are identified, the coordinator gathers the patient data and follows their prognosis until they are discharged from the hospital. The quality of the registry is assessed annually by verifying the items collected via the medical records for 5% of the patients [12]. All of the pediatric trauma patients from 0 to 18 years old who were admitted for at least 1 day between 2001 and 2018 were included in the analysis.

The registry collects comprehensive information about the trauma patients, including the age, gender, mode of transportation, trauma code activation, surgery, and intensive care unit (ICU) admission. In addition, the severity measures are also captured, such as the Glasgow Coma Scale (GCS) score, which is a widely used clinical assessment of consciousness level impairment in response to stimuli. Another severity measure used is the Injury Severity Score (ISS), which is an anatomical measure of the severity that generates a value from 0 to 75 depending on the injury extent in the body regions. The registry also includes the Revised Trauma Score (RTS), which is a physiological scale that is used routinely for all trauma patients when they are first examined, and it includes the GCS score, blood pressure, and respiratory rate [13]. In previous studies, these variables that are collected in the ED have been found to be predictive of trauma outcomes [14]. In SA, emergency transportation is provided by the Saudi Red Crescent Authority (SRCA), which was established approximately 85 years ago. The SRCA serves all of the individuals living in SA at no cost, and it has 78 centers dedicated to serve the Riyadh region [15].

### Independent and dependent variables

The independent variable for this study was the injury mechanism, which was classified as follows: falls, burns, drowning, motor vehicle crashes (MVCs), motorcycle collisions, pedestrian, and intentional injuries (homicides and injuries inflicted on purpose by other persons). Other injury mechanisms, such as suicide (*n* = 2) and those due to mechanical equipment (*n* = 6), were excluded due to their small sample sizes. This study also excluded those patients in which the injury mechanism was classified as “other” (*n* = 812). The primary dependent variable was an extended LOS, which was defined as ≥21 days based on prior research [7, 16, 17].

### Statistical analyses

Stata version 15 for Mac (StataCorp LLC, College Station, TX, USA) was used for all of the statistical analyses. The descriptive statistics were compared with the LOS based on the age, gender, mechanism of injury, mode of transportation, RTS, ISS, GCS score, ICU admission, surgery, trauma team activation, and head injury. The analysis compared the means and standard deviations for the continuous variables and the proportions for the categorical variables. The chi-squared test was used to test for differences among the categorical variables, and the Student’s t test was used for the continuous variables. A *p* value of ≤0.05 was considered to be the cut-off for statistical significance.

Both univariate and multivariate logistic regression analyses were used to evaluate the associations between the injury mechanisms and an extended LOS. The multivariate model was adjusted for age (0–8 as a reference), gender (female as a reference), injury mechanism (falls as a reference), transportation mode (ambulance as a reference), undergoing surgery (binary), ICU admission (binary), and trauma activation (binary). The severity measures were not included in the adjusted model because they were likely to be located on the causal pathway between the injury mechanism and an extended LOS. This study protocol was reviewed and approved by the institutional review board at King Abdullah International Medical Research Center.

## Results

A total of 5563 pediatric patients were included in the analysis. Of those, 774 (14%) patients had extended LOSs. Most of the patients were males (75.8%), and approximately one half of the subjects were children younger than 8 years old (46.3%). Overall, the most common injury mechanism was falling (31.5%), followed by an MVC (28.5%). Approximately one third of the injured patients were admitted to the ICU, and 17.4% of them underwent surgery (Table 1).

**Table 1:** Descriptive characteristics of the pediatric patients admitted to King Abdulaziz Medical City following injuries (2001–2018)

Variable	Short LOS *n* = 4589	Extended LOS *n* = 774	Total *n* = 5563	*P* value
Age (years)				
0–8	2298 (48.0%)	276 (35.6%)	2574 (46.3%)	< 0.001*
9–13	832 (17.7%)	140 (18.1%)	927 (17.5%)	
14–18	1659 (34.6%)	358 (46.25%)	2017 (36.3%)	
Gender				
Male	3681 (76.9%)	588 (76.1%)	4269 (75.8)	0.58*
Female	1108 (23.1%)	186 (24.0%)	1294 (23.26)	
Transportation mode				
Ambulance	1382 (28.9%)	554 (71.6%)	1936 (34.8%)	< 0.001*
Private car	3382 (70.6%)	219 (28.3%)	3601 (64.7%)	
Other	25 (0.5%)	1 (0.1%)	26 (0.5%)	
Injury mechanism				
Burns	572 (12.0)	159 (20.5%)	731 (13.1%)	< 0.001*
Drowning	135 (2.8%)	12 (1.5%)	147 (2.6%)	
Falls	1718 (35.9%)	33 (4.3%)	1751 (31.5%)	
MVCs	1209 (25.2%)	377 (48.7%)	1586 (28.5%)	
Motorcycle crashes	226 (4.7%)	31 (4.0%)	257 (4.6%)	
Pedestrian	716 (15.0%)	147 (19.0%)	863 (15.5%)	
Intentional injuries	213 (4.4%)	15 (1.94%)	228 (4.1%)	
Surgery				
Yes	812(17.0%)	158 (20.4%)	970 (17.4%)	0.02*
No	3977 (83.0%)	606 (79.6%)	4593 (82.6%)	
ICU admission				
Yes	1102 (23.0%)	581 (75.0%)	1682 (30.2%)	< 0.001*
No	3687 (77.0%)	194 (25.1%)	3881 (69.8%)	
Trauma team activation				
Yes	373 (7.8%)	198 (25.6%)	571 (10.3%)	< 0.001*
No	4416 (92.2%)	576 (74.4%)	4992 (90.0%)	
Head injury				
Yes	424 (9.1%)	64 (8.3%)	488 (8.9%)	0.47
No	4255 (90.9%)	710 (91.7%)	4965 (91.0%)	
ISS^a^	6.8 ± 7.5	15.4 ± 9.5	8.0 ± 8.4	**< 0.001**″
GCS score^a^	14.0 ± 2.7	10.4 ± 4.7	13.4 ± 3.3	**< 0.001**″
RTS^a^	11.0 ± 2.6	9.9 ± 2.7	10.8 ± 2.7	**< 0.001**″

*LOS* Length of stay, *MVC* Motor vehicle crash, *ICU* Intensive care unit, *ISS* Injury Severity Score, *GCS* Glasgow Coma Scale, *RTS* Revised Trauma Score

^a^Represented by the mean ± standard deviation

*Chi-squared test

″t test

*P* values in **bold** are significant

The patients with extended LOSs suffered more severe injuries than those with short stays as measured by the ISS (mean scores: 15.4 vs. 6.8, *p* < 0.01), GCS score (mean scores: 10.4 vs. 14.0, *p* < 0.01), and RTS (mean scores: 9.9 vs. 11.0, *p* < 0.01). As expected, the analysis revealed that the patients with extended LOSs were more likely to be admitted to the ICU (75.0% vs. 23.0%, *p* < 0.01) and more likely to undergo surgery (20.4% vs. 17.0%, *p* = 0.02) than the patients with shorter stays.

One half of the patients with extended LOSs were admitted due to MVCs. However, the patients with shorter stays were more likely to be admitted due to falls (35.9% vs. 4.3%, *p* < 0.01). Ambulances were the predominant mode of transportation for the patients with extended LOSs (71.6% vs. 28.9, *p* < 0.01). Unexpectedly, there was no significant difference in the proportion of head injuries between the patients with extended LOSs and those with short stays (Table 1).

The regression analysis identified the injury mechanism as a significant predictor of an extended LOS (Table 2). Those patients with MVC injuries had the highest odds of having an extended LOS than the patients with fall injuries [odds ratio (OR): 16.2, 95% confidence interval (CI): 11.3–23.3]. Additionally, the patients who suffered burns, pedestrian injuries, and motorcycle injuries had, respectively, higher odds of an extended LOS than those with fall injuries (OR: 14.5, 95% CI: 9.8–21.3; OR: 10.7, 95% CI: 7.2–15.7; and OR: 7.1 95% CI: 4.3–11.9; respectively). To a lesser extent, the victims of intentional injuries were 3.6 times more likely to have extended LOSs than those who sustained fall injuries (OR: 3.6, 95% CI: 1.9–6.9) (Table 2, unadjusted model).

**Table 2:** Logistic regression models of the associations between the injury mechanisms and an extended hospital stay

	Unadjusted model		Adjusted model	
	OR	95% CI	OR	95% CI
Age (years)				
0–8			*Reference*	
9–13			1.5	1.1–1.9
14–18			1.7	1.3–2.1
Gender				
Female			*Reference*	
Male			0.7	0.6–0.9
Injury mechanism				
Falls	*Reference*		*Reference*	
MVCs	16.2	11.3–23.3	4.8	3.2–7.1
Motorcycle crashes	7.1	4.3–11.9	3.6	2.1–6.3
Pedestrian	10.7	7.2–15.7	3.7	2.4–5.6
Burns	14.5	9.8–21.3	4.1	2.7–6.3
Drowning	4.6	2.3–9.2	2.3	1.1–4.8
Intentional injuries	3.6	1.9–6.9	2.0	1.0–4.1
Transportation mode				
Ambulance			*Reference*	
Private car			0.3	0.2–0.4
Other			0.2	0.1–1.2
Surgery				
No			*Reference*	
Yes			1.7	1.3–2.1
ICU admission				
No			*Reference*	
Yes			7.4	5.9–9.2
Trauma team activation				
No			*Reference*	
Yes			0.8	0.6–1.1

*OR* Odds ratio, *CI* Confidence interval, *MVC* Motor vehicle crash, *ICU* Intensive care unit

These findings remained significant even after adjusting for the potential confounders. After adjusting for the age, gender, transportation mode, surgery, ICU admission, and trauma team activation, the patients who sustained MVC injuries were almost five times more likely (OR: 4.8, 95% CI: 3.2–7.1) to have extended LOSs than the patients with fall injuries. Moreover, burn, pedestrian, motorcycle, and drowning injuries were still associated with higher odds of an extended LOS (OR: 4.1, 95% CI: 2.7–6.3; OR: 3.7, 95% CI: 2.4–5.6; OR: 3.6, 95% CI: 2.1–6.3; and OR: 2.3, 95% CI: 1.1–4.8; respectively). The analysis also showed that the patients transported via private vehicles were 70% less likely to have an extended LOS than those transported via ambulances (OR: 0.3, 95% CI: 0.2–0.4). Interestingly, males were 30% less likely to have extended LOSs than the females (Table 2, adjusted model).

## Discussion

The results of this study demonstrated that the injury mechanism is an independent predictor of an extended LOS among pediatric trauma patients. Specifically, the MVC injuries were associated with a fivefold increased likelihood of an extended LOS than the fall injuries. Because MVCs are still the leading cause of trauma admission in SA, the results of our study emphasize the fact that they play a significant role in extending pediatric patient hospitalizations [18]. An extended LOS has been linked to increased morbidity, disability, and healthcare utilization during and after hospital discharge [19].

Similar to our results, a study by Evbuomwan et al. found that falling was the most common injury mechanism in northern SA [20]. Because no other studies examined extended LOSs in SA, it was not possible to compare our findings to those from the local literature. However, a study conducted in the US by Burd et al. found that blunt injuries required longer total LOSs when compared with penetrating injuries [19]. Our study also found that males were overrepresented among trauma patients, which is consistent with the national and international literature [18, 21, 22]. This finding may be due to the fact that males are more likely to play unsupervised and engage in risky activities [23]. Public health programs may use these findings to design interventions to specifically reduce preventable injuries among this group.

As expected, severe injuries were significantly associated with extended hospitalizations. MVCs were the second most common cause of severe injuries in US pediatric patients [19]. In SA, MVCs were the leading cause of trauma admission [18]. Approximately 20% of all hospital admissions were the result of MVCs, and many of these patients required extended hospitalizations [24]. One factor that may contribute to the fact that MVCs are associated with severe injuries is the lack of adherence to safety measures, such as seatbelts [25]. In SA, several studies have reported a low restraint use level [26, 27]. In particular, Alsanea et al. reported that the child restraint use prevalence among the Saudi population was 36.6%, and only half of the participants reported consistent use. This is substantially lower than the rates in developed countries like Australia and the US (> 90%) [28, 29]. Clearly, investing in increasing the adherence to child safety restraint use is needed to facilitate reductions in the frequency and severity of MVC injuries.

Although burns were not among the most common injury causes (13.7%), they were associated with a higher risk for an extended hospitalization. This finding was not surprising, because burn victims may require extensive treatment and monitoring. In addition, burn victims are at increased risk for acquiring microorganisms that are highly resistant to antimicrobials, such as *Pseudomonas aeruginosa* [30]. This may lead to extending the LOS to improve recovery, which increases the burden on the healthcare facility. Aside from increased healthcare utilization, burn injuries may lead to long-term psychosocial morbidity due to poor aesthetic and functional outcomes [31]. Unsupervised children may cause burn incidents [32], and to reduce the likelihood of burn injuries, education is a crucial prevention element. Parents play an essential role in prevention, in addition to adequately installed smoke alarms. A previous study by Keswani et al. reported a reduction in mortality in areas in which educational programs about burns were launched [33]. However, further research is needed to evaluate the implementation of public health interventions to reduce burn injuries in SA.

Our results suggested that private transportation was associated with a lower likelihood of an extended LOS. However, it is possible that patients transported by ambulances have more severe injuries than those transported via private vehicles. Approximately two thirds of all of the patients transported to the KAMC came via private transportation, which is similar to the results of a previous local study [34]. This estimate is higher than expected, and it may be explained by a lack of awareness about how to contact emergency services [35]. Further investment is needed to emphasize the role that emergency transportation plays in treating pediatric patients in SA.

The financial costs of extended LOSs place a significant burden on the healthcare system in SA. The estimated cost of the median hospital LOS caused by trauma-related complications can reach up to 150 thousand dollars [36]. Local studies have not investigated the financial burden placed on the government; however, a study in the US had estimated a yearly cost of over 37 billion dollars [37]. However, these are conservative estimates because they only represent the inpatient trauma costs, while the overall costs are likely to be much higher. Therefore, there is an urgent need to implement public health interventions, including the enforcement of road traffic laws, in order to minimize this burden.

Effective prevention strategies are needed to reduce the risk of extended hospital LOSs. SA has had some success in reducing the burden of MVC injuries by implementing public health interventions. For example, a camera system was implemented in 2010, and this was found to be associated with reduced mortality and injury severity [38]. Another recent study by Alghnam et al. examined the impact of introducing a camera system to reduce mobile phone use among drivers [39]. The study found a significant increase in driver compliance with seatbelt use and a reduction in mobile phone violations. Therefore, more strategies that focus on increasing safety measures may yield similar results toward reducing injuries and, ultimately, the hospital LOS.

Currently, SA is implementing its *Vision 2030*, which is a national landmark plan that aims to improve the population health and reduce disabilities. One of the goals of *Vision 2030* is to adopt further measures to ensure traffic safety and to reduce traffic crashes and minimize their tragic consequences [40]. This goal can be achieved when preventive measures are tailored to the population and implemented effectively to promote public health. Physicians and healthcare providers need to play roles in reducing the effects of MVCs on extended LOSs and mortality. Their roles include engaging the public by emphasizing the importance of compliance with child restraints to reduce preventable injuries. Moreover, implementing trauma systems in SA may play a role in reducing the injury mortality and morbidity rates, as reported in other countries [41].

This study was not without limitations. Our research retrospectively evaluated the associations between the injury mechanisms and an extended LOS at a single center. Thus, a generalization to the rest of the country cannot be guaranteed. A nationwide study could provide more conclusive evidence. Another limitation was that we were not able to ensure that all of the potential confounders were adequately controlled, such as the differences in the quality of care. If a patient suffered a medical error, this would influence their likelihood of having an extended LOS, regardless of his or her injury mechanism. Nevertheless, it is unlikely that this affected the treated patients differently, leading to biased results.

Another potential limitation was that we were unable to identify the reasons for the extended LOSs other than those explored in this analysis. An example of this is an extended LOS due to social reasons. Because the healthcare provided at the KAMC is free, the parents of some children, especially those with severe injuries, may refuse to discharge their children because they are hoping for improved health. The other reasons may be related to logistics, such as requiring medical transportation to other healthcare facilities for those not eligible for care at the KAMC. Despite its limitations, this study was the first to explore an important topic that may initiate prevention using evidence-based public health approaches.

## Conclusion

In summary, we found that MVC and burn injuries were associated with a significant burden of the extended LOSs. Prevention is instrumental for reducing healthcare utilization and improving population health. Therefore, these findings call for public health professionals and policymakers to plan and implement preventive measures to reduce the MVC burden. Moreover, public health programs aimed to improve traffic safety for children, including the enforcement of car restraints, are desperately needed to reduce the burden of preventable injuries.

## Data Availability

The study data is available upon request.

## Abbreviations

CI: Confidence interval
ED: Emergency department
GCS: Glasgow Coma Scale
ICU: Intensive care unit
ISS: Injury Severity Score
KAMC: King Abdulaziz Medical City
LOS: Length of stay
MVC: Motor vehicle crash
OR: Odds ratio
RTS: Revised Trauma Score
SA: Saudi Arabia
US: United States
